# Development of a Sesotho health literacy test in a South African context

**DOI:** 10.4102/phcfm.v11i1.1853

**Published:** 2019-04-24

**Authors:** Marianne Reid, Mariette Nel, Ega Janse van Rensburg-Bonthuyzen

**Affiliations:** 1 School of Nursing, Faculty of Health Sciences, University of the Free State, Bloemfontein, South Africa; 2 Department of Biostatistics, Faculty of Health Sciences, University of the Free State, Bloemfontein, South Africa

## Abstract

**Background:**

Research shows that poor health literacy (HL) can be a threat to health and health care. Health literacy is under-researched and poorly understood in developing countries, including South Africa, because of the absence of language and context-specific HL tests.

**Aim:**

The researchers aimed to develop an appropriate HL test for use among South African public health service users with Sesotho as their first language.

**Setting:**

The test was developed in the Free State Province of South Africa, for use among Sesotho speakers.

**Methods:**

Mixed methods were employed to develop the Sesotho Health Literacy Test (SHLT). The process of developing the test was carried out in distinctive methodological steps.

**Results:**

The stepwise process set out by identifying abstracts (*n* = 206) referring to HL tests. Sourcing of HL tests followed a tapered process resulting in the use of 17 HL tests. Elements within a conceptual framework guided HL test item selection (*n* = 47). Two Delphi sessions assisted in reaching consensus regarding final HL test items (*n* = 40). The readability testing of the SHLT tested 4.19 on the Coleman–Liau Index score. A context-suitable and comprehensive SHLT ensued from this work.

**Conclusion:**

The SHLT assessment instrument development creates a platform for HL testing among Sesotho first language speakers in South Africa. The context-sensitive methodology is entrenched in a theoretical framework, distributing HL test items between identified competencies and related skill dimensions and domains. The methodology can be applied to the development of HL tests for other languages and population groups in developing countries.

**Keywords:**

health literacy assessment; primary health care; South Africa; developing countries; public health service; context-sensitive assessment.

## Introduction

Globally, health literacy (HL) is receiving increased attention.^1^ The concept of HL assessment was first introduced more than four decades ago and constitutes an important skill that enables individuals to maintain their health and use health services.^2^ From a public health perspective, poor HL has an impeding impact on health and health care. Research conducted in developed countries during the past two decades highlights an array of associated threats, including deficient knowledge and comprehension of health and illness;^2,3,4^ a diminished ability to use the health care system and manage health-related issues;^5,6,7^ delays in health care-seeking behaviour; less frequent use of preventive health services; difficulty in communicating with health care workers; suboptimal adherence to medication and appointments;^8^ lower health status;^9,10,11,12^ higher morbidity and mortality rates;^13^ more frequent hospitalisation;^14^ increased visits to clinicians and associated elevated health care costs.^15,16^

Health literacy in developing countries, including South Africa, is under-researched and poorly understood. Even though research shows that individuals with poor economic status and low educational levels, who constitute 84% of the South African population, are particularly affected by limited HL,^2,17,18^ information on the status of HL among South Africans is scant, resulting largely from the absence of milieu-appropriate HL tests.^19^ Existing HL tests predominantly originated in developed countries, designed for populations in different socio-economic, cultural and health system milieus, and are typically not suitable for use in South Africa, even if translated into a local language. Translation itself does not guarantee the relevance of existing tests for the health system, health concepts, and health and cultural beliefs of the country and target population for which the translated tests are intended. This limitation was demonstrated in a study conducted in the Eastern Cape Province, South Africa,^20^ among a population with English as a second language using the Rapid Estimate of Adult Literacy in Medicine (REALM) instrument. The study showed that REALM was inappropriate for use in the contextual and cultural milieu, rendering questionable results as a proportion of the participants struggled to comprehend the questions and were unable to complete the test. In order to better understand HL in the country, milieu-appropriate HL tests are needed.

Complicating the development of such tests is the fact that the South African society is particularly diverse. With its 11 official languages, multiplicity of cultures, quadruple burden of disease,^21^ wide-ranging morbidity and mortality profiles,^22^ generally low patient empowerment levels and parallel health care systems, that is, the public and private health care sectors,^23^ the development of appropriate HL tests is not a simple task. One recent study conducted in the Eastern Cape Province reported on a contextually and culturally appropriate item bank of HL questions, namely the Health Literacy Test for Limited Literacy (HELT-LL) for South African patients who make use of the public health care sector.^2,24^ The HELT-LL is in isiXhosa, one of several languages in South Africa.

With this work, the research team aims to develop a HL test for the Basotho ethnic group, with Sesotho as their first language. Although only 8% of households in the country use Sesotho as the first language, the language spoken most often in households in the Free State Province is Sesotho, with nearly three-quarters (71.9%) of the provincial population using this language at home.^17,25^ Developing an HL test for this group will be of considerable value in the quest to better understand HL in the province.

This article is intended to explain the methods and processes employed to develop a context-appropriate Sesotho HL test (SHLT). It is believed that the methods and processes used can be applicable for developing context and culturally appropriate HL tests in other languages.

## Research methods and design

A mixed-methods design was employed to develop the SHLT. The process of developing the test was carried out in eight distinctive steps adapted from Lee and Tsai.^26^

*Step 1* entailed the identification of HL tests. A combination of search words was used (Box 1) to identify abstracts referring to HL tests. EBSCOhost and its included databases served as the electronic platform to identify these tests. Only abstracts in English, as well as studies written in other languages with an English abstract, and publications from 01 Jan. 1995 through 30 April 2017 were included.

BOX 1 Search words used during identification of health literacy tests.

**Table:** 

1. (‘health literacy’ n3 instrument*) or (‘health literacy’ n3 assess*) or (‘health literacy’ n3 test*) or (‘health literacy’ n3 questionnair*) or (‘health literacy’ n3 inventor*) or (‘health literacy’ n3 measuring*) or (‘health literacy’ n3 measurement*) and TI (‘health literac*’ and (client* or patient* or worker* or practitioner* or provider* or professional* doctor* or nurs* or physician*)
2. (‘health literacy’ n3 instrument*) or (‘health literacy’ n3 assess*) or (‘health literacy’ n3 test*) or (‘health literacy’ n3 questionnair*) or (‘health literacy’ n3 inventor*) or (‘health literacy’ n3 measuring*) or (‘health literacy’ n3 measurement*) and TI ‘health literac*’ and (utiliz* or utilis* or usage or implement*)

*Step 2* involved the sourcing of HL tests acting as assessment instruments of HL meeting specific inclusion criteria: (1) complete HL tests or truncated items of tests available in English; (2) obtainable through open access or contact with authors; and (3) tests that measure non–disease specific HL. A follow-up round eliminated HL tests not suitable or not adaptable to the South African public health care system and Basotho culture.

The comprehensive search strategy of these two steps was structured according to the guidelines of the Centre for Reviews and Dissemination^27^ and American Dietetic Association.^28^

*Step 3* encompassed the selection of a HL definition to integrate within the conceptual framework towards the development of the SHLT. A definition that reflected a public health perspective and focused on non–disease specific HL was purposefully selected.

*Step 4* established the conceptual framework forming the theoretical foundation in guiding item development of the SHLT. The framework was drawn from Sørensen et al.’s^29^ integrated model of HL, HL skill dimensions identified by Haun et al.^30^ and the HL definition of Dodson et al.^31^ endorsed by the World Health Organization.

In *Step 5*, HL test items were identified and adapted from sourced HL tests. Where needed, identified test items were adapted to the South African public health care system context and Basotho culture.

**FIGURE 1:**
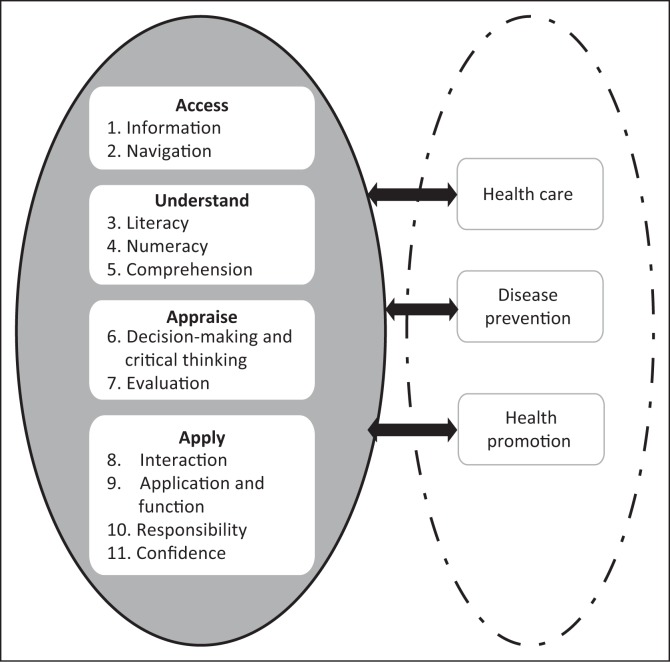
Elements of the conceptual framework forming the theoretical foundation in guiding item development of the Sesotho Health Literacy Test.

During *Step 6*, HL test items were reviewed according to elements in the conceptual framework (Figure 1). The test items were evaluated and modified through a Delphi process.

In *Step* 7, a panel of native Sesotho speakers translated HL test items from English to Sesotho.

*Step 8* allowed testing the readability of test items according to the Coleman–Liau index.

The core research team, composed of a health communication specialist, communication science specialist and a health systems researcher, conducted Steps 1 to 5. Steps 6 and 7 were completed during a 2-day interdisciplinary workshop. Purposefully selected workshop participants (*n* = 11) included health communication specialists, a public health specialist who previously developed HL tests and health promotion representatives from the Free State Department of Health, lecturers in African languages, a nutritionist, a sociologist and health systems researcher. Health communication specialists performed Step 8 after the workshop.

### Ethical considerations

Ethical approval for the research project was obtained from the Health Sciences Research Ethics Committee of the University of the Free State (ECUFS 111/2014).

## Results

### Step 1 – Identify health literacy tests

A total of 286 abstracts referring to HL tests met the inclusion criteria. After excluding abstracts referring to duplicate HL tests (*n* = 80), 206 abstracts remained.

### Step 2 – Source health literacy tests

From the abstracts identified in Step 1 (*n* = 206), an elimination process continued whereby 67 HL tests were sourced and 26 HL tests adhered to the inclusion criteria. After a follow-up process, 17 HL tests (Table 1) remained.

**TABLE 1:** Final list of health literacy tests (*n* = 17) used.

No.	Test†	Abbreviation
1.	All Aspects of Health Literacy Scale	AAHLS^32^
2.	Brief Health Literacy Screener	BHLS^3^
3.	Brief Health Literacy Screening Tool	BRIEF^33^
4.	Communicative and Critical Health Literacy scale	CCHL^34^
5.	eHealth Literacy Scale	eHEALS^35^
6.	Functional, Communicative and Critical Health Literacy scale	FCCHL^36^
7.	General Health Numeracy Test Short Form	GHNT-6^37^
8.	Health Competence Measurement Tool	HCMT^38^
9.	Health Literacy Questionnaire‡	HLQ^39^
10.	Information and Support for Health Actions Questionnaire‡	ISHA-Q^31^
11.	Medication Literacy Assessment in Spanish and English	MedLitRxSE^40^
12.	Nutrition Literacy Assessment Instrument	NLAI^41^
13.	Numeracy Understanding in Medicine Instrument	NUMi^42^
14.	Newest Vital Sign	NVS^43^
15.	Rapid Estimate of Adult Literacy in Medicine – Modified	REALM-M^44^
16.	Short Assessment of Health Literacy – English	SAHL-E^45^
17.	Numeracy Understanding in Medicine Instrument: Short Form	S-NUMi^46^

† , Some of these tests were very similar, different versions of same test or overlapped in parts.

‡ , As full tests were not available, truncated test items were used.

### Step 3 – Select a health literacy definition

An HL definition that was usable within the public health perspective and focused on non–disease specific HL was selected. It is endorsed by the WHO:

Health literacy refers to the personal characteristics and social resources needed for individuals and communities to access, understand, appraise and use information and services to make decisions about health. Health literacy includes the capacity to communicate, assert and enact these decisions.^31^

### Step 4 – Establish a conceptual framework

A conceptual framework (see Figure 1) formed the theoretical underpinning in guiding item development of the SHLT. Four essential competencies of HL include accessing, understanding, appraising and applying health-related information. Each competency is linked to related skill dimensions. Access to health care depends on patients’ ability to find health-related information and navigate health systems. Understanding health information is influenced by patient’s literacy, numeracy and comprehension skills. It is only possible for patients to appraise health information if they are able to critically evaluate health-related decisions. In order to apply these decisions, patients need skills enabling them to communicate on health subjects, apply health information to changing environments, take responsibility for their own health care decisions and express confidence in accomplishing personal and community health. Patients navigate the mentioned competencies and related skill dimensions within three domains, those of health care, disease prevention and health promotion.

### Step 5 – Identify and adapt health literacy test items

Items of the HL tests (*n* = 17) were adapted to the context of the South African public health system and Basotho culture. This process led to the HL test items (*n* = 47) of the SHLT.

Although existing test items were used as examples, it is important to indicate that no test items were used exactly as they appeared in the original tests. All were adapted or changed for purposes of contextualisation. A test item (number 5) from the Numeracy Understanding in Medicine Instrument was contextualised to the South African public health system using a picture of a typical pill-dispensing bag (see Item 12 of the SHLT in Annexure 1). Another example is the adaption of the Newest Vital Sign nutrition label reflecting ice cream information being replaced by a maize meal label. Maize meal is a staple food source of the Basotho (see Item 10 of the SHLT in Annexure 1).

### Step 6 – Review health literacy test items

The identified HL test items (*n* = 47) were reviewed during two separate Delphi sessions during the workshop. The first Delphi session allowed participants to assess test items with reference to understandability and clarity and appropriateness within the South African public health system and Basotho culture. The results for the first Delphi session were as follows. Of the 47 test items, participants deemed 39 (82.9%) to be understandable and clear and 43 (91.5%) appropriate to the context. The items found not understandable or clear (*n* = 8) or appropriate (*n* = 4) were discussed and an attempt made, through group discussion, to adapt items to fit the context of SHLT. Of these, five items could be adapted and were retained, and seven items were eliminated, resulting in a 40-item test. Consensus was considered to be reached when items depicted 50%+ consensus scores.

The second Delphi session (results shown in Tables 2 and 3) created the opportunity for workshop participants to assess the remaining test items’ (*n* = 40) distribution in terms of competencies, skill dimensions and domains as reflected in the conceptual framework. Per illustration, Item 32 in Annexure 1 reflects that ‘HIV can spread through shaking hands’. According to Delphi participants, this item was distributed as assessing understanding as competency, comprehension as a skill dimension and was reflected in all three domains.

**TABLE 2:** Results for Delphi Session 2: Number of items (*n* = 40) indicating consensus for competencies and skill dimensions assessed by Sesotho Health Literacy Test.

Competencies assessed	Skills dimensions	Delphi 2 scores† on 40 remaining items‡	
		*n*	%
Access	Information	10	25
	Navigation	7	18
Understand	Literacy and readability	25	63
	Numeracy	9	23
	Comprehension	28	70
Appraise	Decision-making	23	58
	Evaluation	17	43
Apply	Interaction	4	10
	Application and function	18	25
	Responsibility	13	33
	Confidence	6	15

Note: Test items were further distributed according to domains as contained in the conceptual framework (see Table 3).

† , Consensus was considered to be reached when items depicted 50%+ consensus scores.

‡ , A test item can assess more than one competency or skill dimension.

**TABLE 3:** Results for Delphi Session 2: Number of items (*n* = 40) indicating consensus for domains assessed by Sesotho Health Literacy Test.

Domains assessed	Delphi scores† on 40 remaining items‡	
	*n*	%
Health care	26	65
Disease prevention	22	55
Health promotion	25	70

Note: The English–Sesotho version of the final selected SHLT test items (*n* = 40) is shown in Annexure 1.

† , Consensus was considered to be reached when items depicted 50%+ consensus scores.

‡ , A test item can assess more than one domain.

Scales and options to choose from appear in Annexure 1. Three options were given to choose from for each question including ‘yes’, ‘no’, ‘I don’t know/have not been in such a situation’ or, for example, ‘clinic’, ‘hospital’ and ‘I don’t know’. With word association test items, two-word association options are given, and the third option provides a chance for a respondent to indicate if he or she is unable to make a word association, for example, ‘cough’, ‘weight gain’, ‘I don’t know’. This design decision was based on Dowse’s^19^ publication with reference to limitations of existing HL measures for use in developing countries. Dowse found that the scaled response option is poorly understood or too abstract for use in culturally diverse or low literacy populations and recommended collapsing scales to three points. The test further contained contextually and culturally appropriate pictures of, for example, a syringe, a prescription label or a mealie meal label, to visually operationalise test items measuring health and nutrition literacy or numeracy, which rendered the test more than just a HL screening tool but also a comprehensive performance-based assessment.^34^ The SHLT will be interviewer administered. Scoring of test results, that is, whether a respondent’s HL is adequate, marginal or inadequate, will be set after upcoming psychometric validation of the test.

### Step 7 – Translate test items from English to Sesotho

In Step 7, the test items were translated from English to Sesotho by Sesotho first language workshop participants.

### Step 8 – Test Sesotho readability

The Coleman–Liau index score was 4.19, indicating that the test was suitable for Grade 4 reading level.^47^

## Discussion

This work sought to conceptualise and develop a contextually relevant SHLT for comprehensive assessment of HL in the Sesotho population of the Free State Province of South Africa. A large number of HL tests have been developed over the past 20 plus years, the vast majority by researchers and practitioners in developed countries,^19^ and they are thus poorly suited for use in developing countries. Available tests vary from simple and short HL screening tools^34,35,43^ to complex and comprehensive assessments of HL.^31,37,40^ Tests further vary in terms of the dimensions of HL they measure. Some tests measure general HL^36,37,39^ and others measure literacy pertaining to specific diseases or conditions.^48,49,50^ Very few studies in developing countries are known to have attempted the development of context-relevant HL instruments that encompass multidimensional elements.^2^ Lee et al.^26^ found that the development of new HL assessment instruments is rare. Often, existing instruments are simply translated for use in populations other than the intended target population for which it was developed, or instruments are simply modified slightly to fit another context. These methods, however, fall short in allowing for contextual characteristics of the target population. In this article, we described the methods and processes employed to develop the SHLT – a culturally appropriate and context-specific test of HL in populations that use primarily public health services, such as in South Africa, where 71.4% of patients visit public health services first.^25^

Notwithstanding the fact that more than 60 tests are in existence, Haun et al.^30^ found that important gaps exist, in terms of competencies, skills and dimensions that existing tests assess. The development of the SHLT was guided by specific theoretical and methodological building blocks, which consisted of, firstly, carefully selecting a definition, secondly, following a specific methodological approach and, thirdly, using a strong conceptual framework.

Health literacy is a complex construct and depending on the goal of a study or measurement, definitions vary greatly.^51^ Some definitions are less expansive and refer to obtaining, processing, understanding and using health information.^52^ Others additionally encompass socio-economic, cultural and environmental aspects.^39^ For the purposes of this work, against the background of the complexity and diversity in the South African milieu, we chose to work with a more expansive definition, in order to facilitate sensitivity to not only educational and intellectual characteristics of the target population but also the socio-economic, cultural, morbidity, mortality and health care systems setting. The definition endorsed by the World Health Organization was selected for this work, for its inclusivity of the wider concept of HL. The definition encompasses the personal characteristics and social resources needed for a population to access, understand, appraise and use information to make decisions about their health and to use health care services, as well as the capacity to communicate, assert and enact these decisions.^31^

Osborne et al.^39^ distinguishes between traditional and modern approaches to developing HL tests. Traditional approaches entail using a predefined model,^26^ while modern means a grounded approach is used.^39^ Within our research, a combination of these two approaches was employed. Our work was largely traditional in the sense that literature searches and reviews were employed and focused on developing a theoretically sound conceptual framework using previously published literature on HL. Items and scales in previously developed tests were consulted and used as examples during the development of the SHLT, as can be expected when following the traditional approach. An element of the modern approach was incorporated through ensuring that the lives, that is, the cultural, language, educational and health systems context, of the target population were well understood and reflected in the development of test items. The latter was employed throughout the construction of the SHLT. The authors agree with Osborne et al. that this combination of traditional and modern approaches is effective as well as efficient for the development of culturally and contextually appropriate HL tests, particularly in developing countries.

Statistics South Africa defines literacy as the ability to read and write in at least one language. In an education series published in 2016, Statistics South Africa notes that 15.7% of Sesotho-speaking individuals in South Africa between 25 and 64 years of age are illiterate.^43^ In the Free State, only 80% of individuals 20 years and older have completed primary education (Grade 7). Twenty per cent of the population therefore either has no schooling or has not completed primary education.^25^ Posel^53^ found that most of them fall at the lower end of the literacy scale and have difficulty completing HL tasks and further that there is a gap between grade level and literacy level in South Africa. Despite having completed school, many South Africans remain functionally illiterate.^54^ An HL test pitched at a lower reading level will be more inclusive of a larger group of the target population whose literacy may be on a lower level than the norm for functional literacy. Adult Sesotho-speaking individuals from the Free State Province of South Africa who have completed 4 years of schooling could benefit from the HL test we developed. The methods and processes we employed may have wide application and our experience may be relevant to other developing countries and other languages. The work by Lee and Tsai^26^ similarly supports the use of methodology that is culturally appropriate and country specific.

The authors acknowledge the exclusive focus on patients within the public health sector during the development of the SHLT items. Further research needs to be conducted as to whether the SHLT items are applicable for patients in the private sector. The length of the SHLT renders it unsuitable in its current format to rapidly screen HL, but it could be usable to determine a patient’s HL level. Field-testing of the SHLT has not been completed and is underway. The testing will include cognitive interviews, allowing Sesotho-speaking participants to clarify their understanding of test items. Prior to participants completing the SHLT in order to determine convergent and predictive validity of the test and calibration of the scale, internal validity of the SHLT will also be determined.

## Conclusion

An HL test is effective when it is contextualised to a specific population. The context would include aspects such as language, culture and the health system in which patients receive care. Efficiency not only depends on the content of a HL test but also on the methodological process followed in the development thereof. Test items of the SHLT are distributed between identified competencies, related skill dimensions and domains as part of the theoretical foundation of the SHLT. The contextually sensitive methodology followed can be adapted or used to develop HL tests for other languages and population groups.

## Annexure 1: Sesotho Health Literacy Test items, with English translation

**Table:**

1 I have to ask someone to help me to read information about health.	1. *Ke tlameha ho kopa motho ho nthusa ho bala tlhahiso leseding ka tsa bophelo bo botle*.
Yes No Have not been in such a situation	Eya Tjhee Ha ke so iphumane boemong boo
2 When I need information about health there are sources of health information that are available to me.	2. *Ho na le mehlodi e fumanehang ha ke hloka tlhahiso leseding ka tsa bophelo bo botle*.
Yes No Have not been in such a situation	Eya Tjhee Ha ke so iphumane boemong boo
3 It is easy to get information about health.	3. *Ho bonolo ho fumana tlhahiso leseding ka tsa bophelo bo botle*.
Yes No I don’t know	Eya Tjhee Ha ke tsebe
4 I have someone to talk to about health matters.	4. *Ke na le motho e ke buang le yena ka tsa bophelo bo botle*.
Yes No Have not been in such a situation	Eya Tjhee Ha ke so be boemong bo jwalo
5 I believe my health care provider listens to my problems.	5. *Ke dumela hore mosebeletsi wa ka wa bophelo bo botle o mamela mathata a ka*.
Yes No I don’t know	Eya Tjhee *H*a *ke tsebe*
6 I know where to go to when I have toothache.	6. *Ke tseba moo nka yang ha ke opelwa ke leino*.
Yes No Have not been in such a situation	Eya Tjhee Ha ke so be boemong bo jwalo
7 I know where to get information about losing weight.	7. *Ke tseba moo nka fumanang hlahiso leseding ka ho theola mmele*.
Yes No Have not been in such a situation	Eya Tjhee Ha ke so be boemong bo jwalo
8 If I break my leg, I must go to the	8. *Ha nka robeha leoto, ke tlameha ho ya*
clinic hospital I don’t know	Tliliniking Sepetlele Ha ke tsebe
9 If my brother who stays with me has TB, I must	9. *Ha moholwane wa ka ya dulang le nna a na le TB, ke tlameha ho*
do nothing go to the clinic for TB testing I don’t know	Sa etse letho *H*o *ya tliliniking bakeng sa diteko tsa TB* Ha ke tsebe

**Table:**

**MEALIE MEAL** *Typical Nutritional Information			
**Nutrient**	**Unit**	**Per 100 g serving (uncooked)***	**% Nutrient Reference Values**
Energy	kJ	1380	
Protein Carbohydrate of which total sugar Total Fat Of which:	g g g g	7.6 7.4 2.0 1.7	14%
Saturated fat Trans fat Monounsaturated fat Polyunsaturated fat	g g g g	0.2 0.0 0.3 0.5	
Cholesterol Dietary fibre Total sodium	mg g mg	0 4.1 3	
**Vitamins** Vitamin A(RE) Vitamin B1–Thiamine Vitamin B2–Riboflavin Vitamin B3–Niacin Vitamin B6–Pyridoxine Vitamin B9–Folic acid **Minerals** Iron Zinc	mcg mg mg mg mg mcg mg mg	188 0.3 0.3 3.0 0.4 189 3.7 1.9	21% 25% 15% 19% 24% 47% 21% 17%
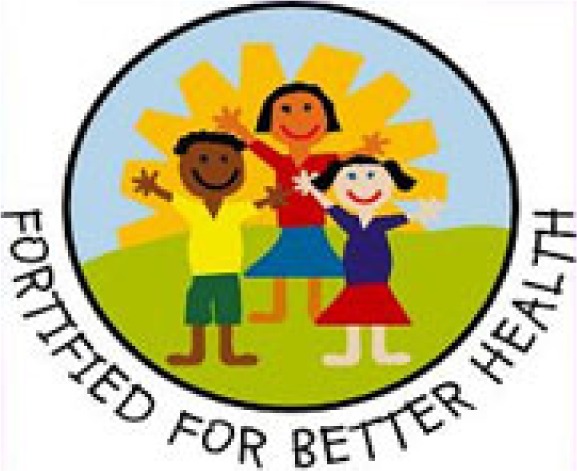

**Table:**

10 If I see this logo on a mieliepap package, it means that the mealie pap contains enough vitamins for me.	10. *Ha ke bona setshwantsho sena mokotlaneng wa phofo ya papa, ho bolela hore papa e na le diaha mmele tse lekaneng.*
Yes No I don’t know	Eya Tjhee Ha ke tsebe

**Table:**

11 Look at the sugar measurements. A cup of sugar equals:	11. *Sheba ditekanyetso tsa tswekere. Kopi ya tswekere e lekana le*
5 mL 250 mL I don’t know	5 mL 250 mL Ha ke tsebe

**Table:**

12 The nurse asked Lerato to take 600 mg of medication a day. Each pill is 200 milligrams (mg). What is the greatest number of pills Lerato should take in 1 day?	12. *Mooki o bolella Lerato ho nwa* 600 mg *ya dipilisi ka letsatsi. Pilisi e le nngwe ke* 200 mg. *Palo e hodimo ya dipilisi eo Lerato a tlamehang ho e fumana ka letsatsi ke bokae*?
3 pills 4 pills I don’t know	*Dipilisi tse* 3 *Dipilisi tse* 4 Ha ke tsebe

**Table:**

13 Look at the instruction on medication bottle. How many times does Tumelo have to take his multivitamin syrup a day?	13. *Sheba ditaelo tse botlolong ya moriana. Tumelo o tlameha ho nwa moriana wa di-aha mmele ha kae ka letsatsi*?
2 times per day 4 times per day I don’t know	2 *ka letsatsi* 4 *ka letsatsi* Ha ke tsebe
14 How many medicine measure-fulls should Tumelo take a day?	14. *Tumelo o tlameha ho nwa tekanyetso tsa moriana tse kae ka letsatsi*?
2 per day 4 per day I don’t know	2 *ka letsatsi* 4 *ka letsatsi* Ha ke tsebe
15 When we read the following words, which option is best associated with the word:	15. *Ha re bala mantswe a latelang, kgetho nyallanang le lentswe leo ke e fe*:
15.1 HIV	15.1 HIV
Disease Witchcraft I don’t know	Bohloko Boloi Ha ke tsebe
15.2 TB	15.2 TB
Cough Weight gain I don’t know	*Ho* ho*hlola* Ho eketseha mmele Ha ke tsebe
15.3 Counselling	15.3 *Thobeho ya maikutlo*
Medication Advice I don’t know	Moriana Keletso Ha ke tsebe
15.4 Overweight	15.4 *Monono*
Sit Walk I don’t know	Dula Tsamaya Ha ke tsebe
15.5 Abuse	15.5 *Hlekefetso*
Kill Hurt I don’t know	Bolaya Lematsa Ha ke tsebe
15.6 Smoking	15.6 *Ho tsuba*
Lungs Kidneys I don’t know	Matshwafo Diphio Ha ke tsebe
15.7 Patient’s rights	15.7 *Ditokelo tsa bakudi*
Money Privacy I don’t know	Tjhelete Lekunutu Ha ke tsebe
16 When reading Sesotho health pamphlets, I always understand all the words on the pamphlet	16. *Ha ke bala dipampitshana tsa bophelo bo botle tsa Sesotho, ke utlwisisa mantswe ohle*
Yes No Have not been in such a situation	Yes No Ha ke so be boemong bo jwalo
17 Your friend is overweight. She does not have money. Appropriate advice you can give her to lose weight is to:	17. *Motswalle wa hao o nonne. Ha a na tjhelete. Keletso e tshwanelehang eo o ka mo fang yona ho theola boima ba mmele ke*:
Go to a gym Take long fast walks I don’t know	Ho lefa ho ya boikwetlisong Ho tsamaya ka potlako nako e telele Ha ke tsebe
18 A person taking a medication for the first time who presents with a skin rash must	18. *Motho a nwang moriana lekgetlo la pele ha a ba le lekgopo o tlameha ho*
Finish the medication Go back to doctor or clinic I don’t know	Ho qeta moriana Ho kgutlela ngakeng/tliliniking Ha ke tsebe
19 A poster states that abuse happens when one person hurts another physically or emotionally. Your neighbour is shouted at daily by her spouse. Do you think your neighbour is abused?	19. *Pampiri ya leboteng e hlalosa hore hlekefetso e etsahala ha motho a utlwisa e mong bohloko mmeleng kapa maikutlo. Moahisane wa hao o omanngwa ka mehla ke molekane wa hae. Na o nahana hore moahisane o a hlekefetswa*?
Yes No I don’t know	Eya Tjhee Ha ke tsebe

**Table:**

**PAIN TABLETS** Per tablet: paracetamol 500 mg; Potassium sorbate 0.12% m/m Sugar free **Warning:** Do not use continuously for longer than 7 days (adults) or 5 days (children) without consulting your doctor. Store below 25°C in a well-closed container protected from light and air. **KEEP OUT OF REACH OF CHILDREN**

**Table:**

20 You have been taking pain pills for 7 days and still have pain. Look at the instructions on the pain tablet label and decide what you have to do:	20. *O nwele dipilisi tsa mahlaba matsatsi a 7 empa o ntse o opelwa. Sheba ditaelo tsena mme o etse qeto ka seo o tlamehang ho se etsa*:
Take 2 pills Go to the doctor or clinic I don’t know	*Enwa dipilisi tse* 2 *E ya ngakeng*/*tliliking* Ha ke tsebe
21 Look at the information on the pain tablet label. May a person who is allergic to aspirin take the medication?	21. *Sheba ditaelo tsena. Motho ya mmele o hananang le Aspirin a ka nwa moriana o latelang*
Yes No I don’t know	Eya Tjhee Ha ke tsebe
22 If you take your first dosage of pain medication at 8 o’clock and the nurse tells you to take the pain medication every 6 h, when can you take your next dosage?	22. *Ha o nwa tekanyetso ya pele ya moriana wa mahlaba ka* 8 *hoseng mme mooki a o bollela ho nwa moriana wa mahlaba ka mora hora tse* 6, *o ka nwa neng tekanyetso e latelang*?
2 o’clock in the afternoon 6 o’clock in the evening I don’t know	Hora ya bobedi motshehare Hora ya botshelela mantsiboya Ha ke tsebe

**Table:**

	***BLOEMFONTEIN CLINICS***	
CLINIC:	**Mopelo Clinic**	
CLINIC TEL:		
TIME:		
NAME OF PATIENT:	**Tefo Molefe**	
ID NUMBER:		
	**BRING THIS CARD EVERY TIME YOU COME TO THE CLINIC**	
***EVIDENCE OF VISIT***		
**DATE**	**TREATMENT**	**SIGNATURE**
1/10/2017	Chronic	XXX
**RETURN** 1/11/2017		

**Table:**

23 Tefo must use medication every day. He only received medication for 2 weeks. The return date is written on the clinic card. Look at the return date and decide if Tefo will have enough medication until his next visit:	23. *Tefo o tlameha ho sebedisa meriana ka mehla. O fumane meriana bakeng sa dibeke tse* 2 *feela. Letsatsi la ho kgutla le ngotswe kareteng ya tliliniki. Sheba letsatsi la ho kgutla mme o etse qeto hore Tefo o tla le meriana e lekaneng ho fihlella letsatsi la hae la tliliniki*:
Yes No I don’t know	Eya Tjhee Ha ke tsebe
24 The nurse recommends you eat a balanced meal. If you decide to eat a plate of spinach, beans and pap, is this a balanced meal?	24. *Mooki oo kgothalletsa ho ja dijo tse itekanetseng. Ha o etsa qeto ho ja sekotlolo sa moroho, dinawa le papa, na dijo tsena di itekanetse na*?
Yes No I don’t know	Eya Tjhee Ha ke tsebe

**Table:**

25 Thabo has to give 2.5 mL of cough syrup to his sister. Choose an option that will indicate that there is 2.5 mL of syrup in the syringe:	25. *Thabo o tlameha ho nwesa kgaitsedi ya hae moriana wa sefuba wa* 2.5 mL. *Etsa kgetho ho bontsha* 2.5 mL *sepeiting*:
Choice A Choice B I don’t know	*Kgetho* A *Kgetho* B Ha ke tsebe

**Table:**

26 If a child’s growth chart looks like this, you will understand it to be that the child is	26. *Ha karete ya ngwana ya kgolo e shebahala tjena, o utlwisisa hore boima ba ngwana bo a*
Gaining weight Losing weight I don’t know	eketseha botheoha Ha ke tsebe
27 I have enough information to manage flu.	27. *Ke na le tlhahiso leseding e lekaneng ka mokgohlane.*
Yes No Have not been in such a situation	Eya Tjhe Ha ke so be boemong bo jwalo
28 My goal is to look healthy.	28. *Takatso ya ka ke ho shebahala ke phetse hantle.*
Yes No Have not thought of this goal	Eya Tjhe Ha ke na takatso e jwalo
29 I make time to exercise.	29. *Ke etsa nako ho ikwetlisa*.
Yes No Have not thought of exercising	Eya Tjhe Ha ke so nahane ho ikwetlisa
30 I try to eat healthy foods.	30. *Ke leka ho ja dijo tse nepahetseng.*
Yes No Have not thought of eating healthy foods	Eya Tjhe Ha ke so nahane ho ja dijo tse itekanetseng
31 Washing hands after using the toilet prevents diseases.	31. *Ho hlapa matsoho ka mora ho sebedisa ntlwana ho thibela mafu.*
Yes No I don’t know	Eya Tjhe Ha ke tsebe
32 HIV can spread through shaking hands.	32. *HIV e ka ata ka ho tshwarana ka matsoho.*
Yes No I don’t know	Eya Tjhe Ha ke tsebe
33 Enough sleep is needed to keep one healthy.	33. *Hore o phele hantle o hloka boroko bo lekaneng.*
Yes No I don’t know	Eya Tjhe Ha ke tsebe
34 You do not understand what a health care worker said. You do which of the following?	34. *Ha o utlwisisi seo mosebeletsi wa bophelo bo botle a se buileng. O etsa eng ho tse latelang*?
Ask the health care worker to explain again Keep quiet Have not been in such a situation	Botsa mosebeletsi wa bophelo ho hlalosa hape Thola Ha ke so be boemong bo jwalo

## References

[1] Lee S-YD, Bender DE, Ruiz RE, Cho YI Development of an easy-to-use Spanish health literacy test. Health Serv Res. 2006;41(4):1392–1412. 10.1111/j.1475-6773.2006.00532.x 16899014 PMC1797080

[2] Marimwe C, Dowse R Development of an item bank of health literacy questions appropriate for limited literacy public sector patients in South Africa. J Commun Healthc. 2017;10(4):273–284. 10.1080/17538068.2017.1380577

[3] Chew LD, Bradley KA, Boyko EJ Brief questions to identify patients with inadequate health literacy. Fam Med. 2004;36(8):588–594. 15343421

[4] Cho YI, Lee S-YD, Aronzulla AM, Crittenden KS Effects of health literacy on health status and health service utilization amongst the elderly. Soc Sci Med. 2008;66:1809–1816. 10.1016/j.socscimed.2008.01.003 18295949

[5] Davis TC, Wolf MS, Bass PF, et al Low literacy impairs comprehension of prescription drug warning labels. J Gen Intern Med. 2006;21:847–851. 10.1111/j.1525-1497.2006.00529.x 16881945 PMC1831578

[6] Kalichman SC, Benotsch E, Sarez T, Catz S, Miller J, Rompa D Health literacy and health-related knowledge among persons living with HIV/AIDS. Am J Prev Med. 2000;18(4):325–331. 10.1016/S0749-3797(00)00121-5 10788736

[7] Hunt S, Dowse R, La Rose C Health literacy assessment: Relexicalising a US test for a South African population. Southern Afr Ling Appl Lang Stud. 2008;26(2):267–281. 10.2989/SALALS.2008.26.2.7.571

[8] Foulk D, Carroll P, Wood SN Addressing health literacy: A description of the intersection of functional literacy and health care. Am J Health Stud [serial online]. 2001;17(1):7–14 [cited 2017 Dec 5]. Available from: http://www.biomedsearch.com/article/Addressing-health-literacy-description-intersection/83662680.html

[9] Tessler Lindau S, Basu A, Leitsch SA Health literacy as a predictor of follow-up after an abnormal Pap smear: A prospective study. J Gen Intern Med. 2006;21:829–834. 10.1111/j.1525-1497.2006.00534.x 16881942 PMC1831576

[10] Williams MV, Baker DW, Parker RM, Nurss JR Relationship of functional health literacy to patients’ knowledge of their chronic disease: A study of patients with hypertension and diabetes. Arch Intern Med. 1998;158(2):166–172. 10.1001/archinte.158.2.166 9448555

[11] Gazmararian JA, Baker DW, Williams MV, et al Health literacy among Medicare enrolees in a managed care organisation. JAMA. 1999;281(6):545–551. 10.1001/jama.281.6.545 10022111

[12] Sudore RL, Yaffe K, Satterfield S, et al Limited literacy and mortality in the elderly: The health, aging, and body composition study. J Gen Intern Med. 2006;21(8):806–812. 10.1111/j.1525-1497.2006.00539.x 16881938 PMC1831586

[13] Bostock S, Steptoe A Association between low functional health literacy and mortality in older adults: Longitudinal cohort study BMJ 2012;344:e1602 10.1136/bmj.e1602 PMC3307807 22422872

[14] Baker DW, Parker RM, Williams MC, Clark WS, Nurss J Health literacy and the risk of hospital admission. J Gen Intern Med. 1998;13(12):791–798. 10.1046/j.1525-1497.1998.00242.x 9844076 PMC1497036

[15] Weiss BD, Blanchard JS, McGee DL, et al Illiteracy among Medicaid recipients and its relationship to health care costs. J Health Care Poor Underserved. 1994;5(2):99–111. 10.1353/hpu.2010.0272 8043732

[16] Tsai TI, Lee S-YD, Tsai YW, Kuo KN Methodology and validation of health literacy scale development in Taiwan. J Health Commun. 2011;16(1):50–61. 10.1080/10810730.2010.529488 21058141

[17] Statistics South Africa (StatsSA) Community survey 2016 [homepage on the Internet]. Electronic citation. 2016 [cited 2018 Feb 9]. Available from: http://www.statssa.gov.za

[18] Leon N, Mabope R The private health sector In: South African health review. Durban: HST, 2005; pp. 34–43.

[19] Dowse R The limitations of current health literacy measures for use in developing countries. J Commun Healthc. 2016;9(1):4–6. 10.1080/17538068.2016.1147742

[20] Dowse R, Lecoko L, Ehlers MS Applicability of the REALM health literacy test to an English second-language South African population. Pharm World Sci. 2010;32(4):464–471. 10.1007/s11096-010-9392-y 20490680

[21] Schneider M, Baron E, Breuer E, et al Integrating mental health into South Africa’s health system: Current status and way forward In: Padarath A, King J, Mackie E, Casciola J, editors. South African Health Review. Durban: HST, 2016; p. 153–164.

[22] Scott V, Schaay N, Schneider H, Sanders D Addressing social determinants of health in South Africa: The journey continues In: Padarath A, Barron P, editors. South African Health Review. Durban: HST, 2017; p. 77–88.

[23] Young M Private vs. public healthcare in South Africa [unpublished thesis] Traverse City, MI: Western Michigan University, 2016; Paper 2741 [cited 2017 Dec 5]. Available from: http://scholarworks.wmich.edu/honors_theses

[24] Marimwe C Development and validation of a health literacy measure for limited literacy public sector patients in South Africa [unpublished thesis] Grahamstown: Rhodes University, 2018.

[25] Statistics South Africa (StatsSA) General household survey 2016 [homepage on the Internet]. Electronic citation. 2016 [cited 2018 Feb 9]. Available from: http://www.statssa.gov.za

[26] Lee S-YD, Tsai TI Health literacy assessment In: Kim DY, Dearing JW, editors. Health communication research measures. New York: Peter Lang, 2015; p. 57–64.

[27] Centre for Reviews and Dissemination (CRD) Systematic reviews: CRDs guidance for undertaking reviews in health care. Layerthorpe: University of York; 2009.

[28] American Dietetic Association Evidence analysis manual: Steps in the Academy evidence analysis process. Chicago, IL: Academy of Nutrition and Dietetics; 2016.

[29] Sørensen K, Van den Broucke S, Fullam J, et al. Health literacy and public health: A systematic review and integration of definitions and models BMC Public Health 2012;12:80 10.1186/1471-2458-12-80 PMC3292515 22276600

[30] Haun JN, Valerio MA, McCormack LA, Sørensen K, Paasche-Orlow MK Health literacy measurement: An inventory and descriptive summary of 51 instruments. J Health Commun. 2014;19(2):302–333. 10.1080/10810730.2014.936571 25315600

[31] Dodson S, Good S, Osborne RH Health literacy toolkit for low- and middle-income countries: A series of information sheets to empower communities and strengthen health systems [homepage on the Internet] New Delhi: World Health Organization, Regional Office for South-East Asia 2015 [cited 2017 Dec 5]. Available from: http://www.searo.who.int/entity/healthpromotion/documents/hl_tookit/en/

[32] Chinn D, McCarthy C All Aspects of Health Literacy Scale (AAHLS): Developing a tool to measure functional, communicative and critical health literacy in primary healthcare settings. Patient Educ Couns. 2013;90(2):247–253. 10.1016/j.pec.2012.10.019 23206659

[33] Haun J, Luther S, Dodd V, Donaldson P Measurement variation across health literacy assessments: Implications for assessment selection in research and practice. J Health Commun. 2012;17(3):141–159. 10.1080/10810730.2012.712615 23030567

[34] Ishikawa H, Nomura K, Sato M, Yano E Developing a measure of communicative and critical health literacy: A pilot study of Japanese office workers. Health Promot Int. 2008;23(3):269–274. 10.1093/heapro/dan17 18515303

[35] Norman CD, Skinner HA eHEALS: The eHealth Literacy Scale. J Med Internet Res. 2006;8(4):e27 10.2196/jmir.8.4.e27 17213046 PMC1794004

[36] Ishikawa H, Takeuchi T, Yano E Measuring functional, communicative, and critical health literacy among diabetic patients. Diabetes Care. 2008;31(5):874–879. 10.2337/dc07-1932 18299446

[37] Osborn CY, Wallston KA, Shpigel A, Cavanaugh K, Kripalani S, Rothman RL Development and validation of the General Health Numeracy Test (GHNT). Patient Educ Couns. 2013;91(3):350–356. 10.1016/j.pec.2013.01.001 23433635 PMC3644342

[38] Mbuagbaw L, Momnougui RCB, Thabane L, Ongolo-Zogo P The health competence measurement tool (HCMT): Developing a new scale to measure self-rated ‘health competence’. Patient Educ Couns. 2014;97:396–402. 10.1016/j.pec.2014.09.013 25308953

[39] Osborne RH, Batterham RW, Elsworth GR, Hawkins M, Buchbinder R The grounded psychometric development and initial validation of the Health Literacy Questionnaire (HLQ) BMC Public Health 2013;13:658 10.1186/1471-2458-13-658 PMC3718659 23855504

[40] Sauceda JA, Loya AM, Sias JJ, Taylor T, Wiebe JS, Rivera JO Medication literacy in Spanish and English: Psychometric evaluation of a new assessment tool. J Am Pharm Assoc. 2012;52(6):e231–e240. 10.1331/JAPhA.2012.11264 23229985

[41] Gibbs HD Nutrition Literacy: Foundations and development of an instrument for assessment [homepage on the Internet] [Faculty scholarship: Family and Consumer Science Paper 1]. Bourbonnais: Olivet Nazarene University; 2012 [cited 2017 Dec 5]. Available from: http://digitalcommons.olivet.edu/facs_facp/1

[42] Schapira MM, Walker CM, Cappaert KJ, et al The numeracy understanding in medicine instrument (NUMi): A measure of health numeracy developed using item response theory. Med Decis Making. 2012;32(6):851–865. 10.1177/0272989X12447239 22635285 PMC4162626

[43] Weiss BD, Mays MZ, Martz W, et al Quick assessment of literacy in primary care: The newest vital sign. Ann Fam Med. 2005;3(6):514–522. 10.1370/afm.405 16338915 PMC1466931

[44] La Rose CM Exploring health literacy assessment: The relexicalisation of a health literacy test from the US for application in a South African population [unpublished thesis] Grahamstown: Rhodes University; 2018 [cited 2017 Dec 5]. Available from: https://core.ac.uk/download/pdf/145030973.pdf

[45] Lee S-YD, Stucky BD, Lee JY, Rozier RG, Bender DE Short assessment of health literacy-Spanish and English: A comparable test of health literacy for Spanish and English speakers. Health Serv Res. 2010;45(4):1105–1120. 10.1111/j.1475-6773.2010.01119.x 20500222 PMC2910571

[46] Schapira MM, Walker CM, Miller T, et al Development and validation of the numeracy understanding in medicine instrument short form. J Health Commun. 2014;19(2):240–253. 10.1080/10810730.2014.933916 25315596 PMC4201377

[47] Krige M, Reid M A pilot investigation into the readability of Sesotho health information pamphlets. Communitas. 2017;22(1):113–123. 10.18820/24150525/Comm.v22.9

[48] Nath CR, Sylvester ST, Yasek V, Gunel E Development and validation of a literacy assessment tool for persons with diabetes. Diabetes Educ. 2001;27(6):857–864. 10.1177/014572170102700611 12211925

[49] Tique JA, Howard LM, Gaveta S, et al Measuring health literacy among adults with HIV infection in Mozambique: Development and validation of the HIV literacy test. AIDS Behav. 2017;21(3):822–832. 10.1007/s10461-016-1348-3 26961538 PMC5306223

[50] Griffin JM, Partin MR, Noorbaloochi S, et al Variation in estimates of limited health literacy by assessment instruments and non-response bias. J Gen Intern Med. 2010;25:675–681. 10.1007/s11606-010-1304-2 20224964 PMC2881963

[51] Berkman ND, Davis TC, McCormack L Health literacy: What is it? J Health Commun. 2010;15(2):9–19. 10.1080/10810730.2010.499985 20845189

[52] Institute of Medicine (US) Committee on Health Literacy Health literacy: A prescription to end confusion [homepage on the Internet] Washington, DC: National Academies Press, USA; 2004 [cited 2018 Mar 12]. Available from: https://www.ncbi.nlm.nih.gov/books/NBK216032/doi:10.17226/10883 25009856

[53] Posel D Adult literacy rates in South Africa: A comparison of different measures. Language Matters. 2011;42(1):39–49. 10.1080/10228195.2011.571703

[54] Aitcheson J Proxies and perplexities: What is the current state of adult (il)literacy in South Africa? J Educ. 2016;66:111–138.

